# Supplementary material for: Deletion of wfs1 Impairs Oligodendrocyte Precursor Cells Dorsal Distribution and Myelination Through the wfs1-hmgcs1 Axis in Zebrafish
Source: Biology (Basel). 2026 Aug 16;15(16):1402. doi: 10.3390/biology15161402 (PMC13510151; doi:10.3390/biology15161402)
Supplement: Supplementary file 1 [file biology-15-01402-s001.zip › File S1-Transcriptome sequencing data and differentially expressed genes (DEGs) in wfs1-- zebrafish at 5 dpf/2.KEGG/wfs1KOvsWT/UP/src/dre04371.html]

dre04371


- dre:323509

- Up regulated genes

323509\_1(2.61236898874666)
- dre:100331581

- Up regulated genes

100331581(2.2819785165305)
- dre:335655

- Up regulated genes

335655\_1(1.29441752237038)

- dre:117508

- Up regulated genes

117508\_1(1.34887174636376) 117508(1.41697225185209)

- dre:321449

- Up regulated genes

321449(1.55311962992127)

- dre:677750

- Up regulated genes

677750(1.37913343503877)

- dre:568726

- Up regulated genes

568726(1.14419230813088)
- dre:560807

- Up regulated genes

560807(1.71247301071773)

- dre:557486

- Up regulated genes

557486\_1(1.35679787207179)

- dre:117508

- Up regulated genes

117508\_1(1.34887174636376) 117508(1.41697225185209)

- dre:100331581

- Up regulated genes

100331581(2.2819785165305)
- dre:335655

- Up regulated genes

335655\_1(1.29441752237038)

- dre:30498

- Up regulated genes

30498\_1(7.44988431851508)

- dre:100331581

- Up regulated genes

100331581(2.2819785165305)
- dre:335655

- Up regulated genes

335655\_1(1.29441752237038)

- dre:192322

- Up regulated genes

336121(1.42904963424916)

Close
